# Rewilding and restoring nature in a changing world

**DOI:** 10.1371/journal.pone.0254249

**Published:** 2021-07-14

**Authors:** Benis N. Egoh, Charity Nyelele, Karen D. Holl, James M. Bullock, Steve Carver, Christopher J. Sandom

**Affiliations:** 1 Department of Earth System Science, University of California Irvine, Irvine, California, United States of America; 2 Environmental Studies Department, University of California, Santa Cruz, California, United States of America; 3 UK Centre for Ecology & Hydrology, Wallingford, Oxfordshire, United Kingdom; 4 School of Geography, University of Leeds, Leeds, United Kingdom; 5 School of Life Sciences, University of Sussex, Brighton, United Kingdom; Chinese Academy of Forestry, CHINA

Increased anthropogenic pressure, invasive alien species and climate change, among other factors, continue to negatively impact and degrade the planet’s ecosystems and natural environment. As nature declines at alarming rates, the loss of biodiversity is not only a huge concern, but it also undermines the many ecological, social, human health and wellbeing benefits nature provides us. Numerous reports, including those from the Intergovernmental Science-Policy Platform on Biodiversity and Ecosystem Services (IPBES, https://www.ipbes.net/), have documented this unprecedented decline in nature across space and time. For example, the 2019 IPBES global assessment report on biodiversity and ecosystem services shows that 75% of the global land surface has been significantly altered, 66% of the ocean area is experiencing increasing cumulative impacts, and over 85% of wetland area has been lost (Brondizio et al. [1]). All the recent IPBES reports from global to regional scales and the Millennium Ecosystem Assessment of 2005 (Reid et al. [2]), point to one thing: the urgency for us to act to save nature and humankind. Ecological restoration has emerged as a powerful approach to combat degradation in land and water, mitigate climate change, and restore lost biodiversity and key ecosystem functions and services. In June this year (2021), the United Nations (UN) is launching the Decade on Ecosystem Restoration (https://www.decadeonrestoration.org/), an ambitious program to trigger a global movement for restoring the world’s ecosystems. In line with that, PLOS ONE commissioned this Collection on Rewilding and Restoration. This is consistent with the year’s Earth Day theme, "Restore Our Earth”, which calls on everyone to be a part of the change and to focus on natural processes, emerging green technologies and innovative thinking that can restore the world’s ecosystems.

When PLOS ONE launched this Rewilding and Restoration collection, we were asked to identify exciting advances and emerging trends observed recently in the areas of rewilding and restoration. We highlight: 1) increasing recognition of the value of restoration in ecosystems worldwide, particularly in a time of rapid global environmental change; 2) understanding and incorporating benefits and beneficiaries in supporting and financing restoration initiatives; 3) exploring the theoretical underpinning for the importance of ‘megabiota’–the largest plants and animals–for driving biosphere scale processes such as ecosystem total biomass, resource flows and fertility; and 4) showcasing success stories on how rewilding nature in the developing world is reversing the impact of invasive species (https://everyone.plos.org/2020/08/28/taking-a-walk-on-the-wild-side/). The broad range of publications in this Collection cover all these areas and much more, making it one of the most exciting collections on rewilding and restoring nature in recent times. The two main themes that emerge from the collection are related to restoration success stories (>40%) and best practices in restoration around the globe (>30%). The selected studies in this Collection, which cover six continents and at least 13 countries, were carried out in diverse settings and contexts, such as marine, fresh water and terrestrial habitats including forests and grasslands, rivers and coastal areas, woodlands, wetlands, and mountains (e.g., Sansupa et al. [3], Broughton et al. [4], Schulz et al. [5], Ndangalasi et al. [6]). Features of interest included in this Collection span from bacteria through large vertebrates (e.g., wild dogs, elephants) to ecosystems and their functions. These articles also showcase a range of methodological approaches from a series of small-scale field experiments (Wasson et al. [7]), wildlife tracking and remote sensing (Mata et al. [8]), and large-scale models to predict restoration outcomes (D’Acunto et al. [9]).

This rich collection from PLOS ONE addresses a range of related and interesting issues: 1) Different restoration approaches, from passive rewilding to active target driven restoration, are needed to achieve different restoration goals in different circumstances. 2) Nature is complex and context dependent and so diverse approaches to restoration will help ensure different taxonomic groups and ecosystem functions and services are supported. 3) Developing and recording best practice for different restoration approaches will greatly aid the achievement of restoration aims. 4) Measuring restoration success needs comprehensive, multi-dimensional, and quantifiable metrics to account for potentially complex trade-offs. 5) Arguments for restoration based on ecocentric and nature’s contribution to people both have merit and appeal to different audiences, but it should not be assumed goals derived from these different ways of thinking will be aligned. This is a diverse collection of restoration and rewilding research, and that diversity neatly reflects the diverse approaches and goals needed for restoration to be successful.

The articles in this issue discuss case studies that span a continuum of restoration interventions from removing anthropogenic disturbance and allowing the ecosystem to regenerate naturally (i.e., passive restoration or rewilding) to intensive interventions with ongoing management. For example, Broughton et al. [4] found that secondary woodlands in England that were adjacent to ancient woodlands recovered naturally over a period of a few decades. Díaz-García et al. [10] compared recovery of amphibians, ants, and dung beetles in naturally regenerating and actively planted tropical forests in Mexico; they found that passive and active restoration approaches were similarly effective in restoring species richness of all guilds, but that forest specialists were enhanced through active planting. In contrast, other studies show that intensive anthropogenic interventions such as transplanting corals (Ferse et al. [11]), or controlling invasive species and reintroducing fauna (Roberts et al. [12]) are necessary to facilitate recovery. The diversity of responses reported highlights the need to tailor restoration strategies to the local ecosystem type, the species of interest, and the level of prior disturbance.

Similarly, studies in this collection demonstrate complex interactions between wild and domestic herbivory, controls on grazing intensity and spatial ecological variables, making generalizations difficult and stressing the need for context-specific studies and understanding to guide management of disturbance regimes. One study in African savanna (Young et al. [13]) explores the impact of grazing on biodiversity and shows that plots protected from herbivory by large wild herbivores for the past 25 years have developed a rich diversity of woody vegetation species which could disappear upon rewilding depending on level of predation and associated behavioral patterns. However, they also show that individuals of the dominant tree species in this system, *Acacia drepanolobium*, greatly reduce their defense in the absence of browsers; hence the sudden arrival of these herbivores resulted in far greater elephant damage than for conspecifics in adjacent plots that had been continually exposed to herbivory. Similarly, Peacock et al. [14] suggests that cattle negatively impact regeneration of gallery forests in Bolivia and alter both the structure and composition of the shrub and ground layers with potential consequences for the diversity and abundance of wildlife. Previous studies including Hanke et al. [15] have shown increases in species diversity and ecological functioning with grazing. These results suggest that the impact of grazing on ecosystems, species and ecosystem functioning depends on the system, the grazing species, and their numbers, and overall carrying capacity.

Several best practices are highlighted in the Collection. Larson et al. [16] created a model to determine an “optimal maximum distance” that would maximize availability of native prairie seed in the midwestern United States (US) from commercial sources while minimizing the risk of novel invasive weeds via contamination. Pedrini et al. [17] test seed pretreatment methods to enhance vegetation establishment from direct seeding and illustrate how a range of life stage transitions including germination, emergence and survival of native grass species used in restoration programs can be improved by seed coating with salicylic acid. Roon et al. [18] used a before-after-control-impact experiment across three stream networks in the northwestern to provide guidance on riparian thinning to provide optimal stream habitat. These best practices are key in our ability to replicate in different places and achieving restoration success.

Determining the success of ongoing restoration efforts is crucial to assessing management actions but requires comprehensive, multi-dimensional, and quantifiable metrics and approaches consistent with restoration goals. Despite the plethora of restoration projects around the world, it is only now that we are beginning to understand whether the restoration goals have been met and what trade-offs exist (Mugwedi et al. [19]). The importance of measuring restoration outcomes against clearly specified goals and objectives cannot be overemphasized, as shown in a recent restoration study in China that aimed to improve carbon storage through tree planting but has severely depleted water resources (Zhao et al. [20]). Similarly, Valach et al. [21] show that productive wetlands restored for carbon sequestration quickly become net carbon dioxide (CO_2_) sinks although the trade-offs need to be further assessed. In their study exploring restoration success in South Africa, del Río et al. [22] improve our understanding on how techniques such as remote sensing can be used to measure restoration success.

As shown in this Collection and in other studies, trade-offs in restoration efforts are not uncommon and ultimately, restoration is successful when we can achieve restoration goals while minimizing trade-offs. The successful stories from the restoration interventions across different habitats and species showcased in the Collection (e.g., Sansupa et al. [3], Roon et al. [18], Valach et al. [21], Bouley et al. [23]) are a valuable addition to the science needed to advocate for restoration as a pathway to the recovery of previously degraded, damaged, or destroyed ecosystems. Reporting successful restoration outcomes can help increase buy-in for further restoration projects and increase funding availability for such projects. However, such buy-in can only occur if stakeholders are interested in the set restoration goals. For example, the need for climate mitigation has been used to justify several restoration programs around the world (Alexander et al. [24], Griscom et al. [25]). In this Collection, Matzek et al. [26] ask whether including ecosystem services as a restoration goal will engage a different set of values and attitudes than biodiversity protection alone. They found that support for habitat restoration is generally based on ecocentric values and attitudes, but that positive associations between pro-environmental behavior and egoistic values emerge when emphasis is placed on ecosystem service outcomes. They emphasize the notion that the ecosystem services concept garners non-traditional backers and broadens the appeal of ecological restoration as it is seen as a means of improving human well-being. Nevertheless, several studies (Bullock et al. [27], Egoh et al. [28], Newton et al. [29]) have shown that there can be trade-offs between biodiversity and services during restoration and among different services, so restoration aims need to be clear rather than assuming win-win outcomes. Indeed, previous studies including Berry et al. [30] have suggested that a broad spectrum of perspectives on biodiversity conservation exist and should be used as arguments for conservation actions, from intrinsic to utilitarian values. In their analysis, the main differences between types of arguments appeared to result from the espousal of ecocentric or anthropocentric viewpoints, rather than from differences between the various stakeholder groups. This suggests that to promote restoration goals, a broad range of restoration goals are needed, including those that are more anthropocentric such as economic development.

While the positive impacts of ecological restoration on biodiversity are well established, less evidence is available regarding its impacts on economic development and employment. Although restoration efforts centered around economic development in Africa, such as the Working for Water Project and eThekwini forest restoration project in South Africa have generated strong support from government, not many such initiatives exist in other parts of the world (Mugwedi et al. [19]). In this collection, Newton et al. [29] examine the impacts of restoration on economic development and employment. They conclude that landscape-scale restoration or rewilding of agricultural land can potentially increase the contribution of farmland to economic development and employment, by increasing flows of multiple ecosystem services to the many economic sectors that depend on them. Indeed, restoration has contributed to the economy in many parts of the world leading to the framing of the term “restoration economy” or “green economy” which is now commonly used in the restoration literature (Bek et al. [31], Formosa et al. [32]). A recent report by Dasgupta [33] states that “our economies are embedded within Nature, not external to it”. While we are all looking forward to the UN Decade on Ecosystem Restoration launching this year, the uptake of restoration projects will depend on financing. Generating funds to support and sustain restoration projects is one of the biggest challenges facing restoration activities worldwide (FAO and Global Mechanism of the UNCCD [34]). The inclusion of a broad range of goals that span from biodiversity to anthropocentric goals such as those related to benefits of nature’s contribution to people to those that are purely development such as job creation may be the way forward.
